# Missed Opportunities for Timely Diagnosis and Effective Therapeutic Management of Prolonged Fever: A Case Study of Confirmed Lassa Fever in N’Zérékoré, Guinea, 2022

**DOI:** 10.1155/crdi/2672939

**Published:** 2026-06-24

**Authors:** Ibrahima Sory Cherif, Esme Marie Laure Essis, Mamadou Alpha Diallo, Loukou Léandre Konan, Aly Antoine Kamano, Djoukou Olga Denise Kpebo, Seydou Dia, Mamadou Oury Baldé, Sory Condé, Fatoumata Cherif, Momory Millimono, Fanta Mady Kouyaté, Angelo Loua, Lila Condé

**Affiliations:** ^1^ World Health Organization Office, BP 817, Conakry, Guinea; ^2^ Ministry of Health and Public Hygiene, BP 234, Conakry, Guinea; ^3^ Center for Research and Studies on Population, Health Policies and Systems, National Institute of Public Health, BPV 47 CI-AB. IV93. 14/CI.AB. 110, Abidjan, Côte d’Ivoire, insp.mx; ^4^ Clinical Research Center on Neglected Tropical Diseases and Associated Pathologies, National Institute of Public Health, BPV 47 CI-AB. IV93. 14/CI.AB. 110, Abidjan, Côte d’Ivoire, insp.mx; ^5^ Maternal and Child Health Service, National Institute of Public Health, BPV 47 CI-AB. IV93. 14/CI.AB. 110, Abidjan, Côte d’Ivoire, insp.mx; ^6^ Department of Public Health and Biostatistics, UFR of Medical Sciences, Félix Houphouët Boigny University, 01 BP V34 Réf.ECI554 CI-AB. IV93. 14/CI.AB. 110, Abidjan, Côte d’Ivoire; ^7^ National Health Security Agency, BP 787, Conakry, Guinea

**Keywords:** case report, contact case, Guinea, Lassa fever, lessons learned, N’Zérékoré, outbreak, viral hemorrhagic diseases, West Africa

## Abstract

**Background:**

Lassa fever is a potentially fatal viral hemorrhagic disease caused by a ribonucleic acid (RNA) virus of the *Arenaviridae* family, endemic in West Africa. It is highly virulent and contagious in several countries, with a nonspecific clinical presentation that poses a major public health challenge. An in‐depth investigative survey was conducted in N’Zérékoré to identify the transmission chain of a confirmed case of Lassa fever.

**Case Presentation:**

This study reports a confirmed case of Lassa fever diagnosed 13 days after the onset of persistent fever. The disease was suspected at the regional hospital of N’Zérékoré following the worsening of the initial clinical picture, characterized by the onset of hemorrhagic manifestations and failure of antibiotic therapy. Confirmation of Lassa virus infection by reverse transcriptase–polymerase chain reaction (RT‐PCR) on a blood sample was obtained a day prior to the patient’s death. The patient did not receive ribavirin treatment and remained on the same antibiotic regimen (ceftriaxone) from the onset of fever, combined with dexamethasone, omeprazole, and a unit of blood transfusion.

**Conclusion:**

The therapeutic pathway of this confirmed Lassa fever case in N’Zérékoré highlights the need for improved management of viral hemorrhagic fevers and consideration of differential diagnoses when broad‐spectrum antibiotic therapy fails.

## 1. Introduction

Lassa fever remains a persistent global health threat, accounting for approximately half a million cases annually in West Africa. Lassa fever is an acute viral hemorrhagic zoonotic disease transmitted to humans through contact with food or household items contaminated by urine or feces from infected *Mastomys natalensis* rodents [1]. Human‐to‐human transmission occurs through direct contact with infected blood or bodily secretions, particularly in the laboratory and hospital settings, highlighting weaknesses in infection prevention and control measures [1–4]. This disease is caused by a virus belonging to the Arenaviridae family, circulating in West Africa since its discovery in 1969 in the village of Lassa, northeastern Nigeria [3]. It is endemic in Guinea, Liberia, Nigeria, Benin, and Sierra Leone, with sporadic cases reported in Côte d’Ivoire, and Ghana [3, 5].

Every year, several hundred thousand cases of Lassa virus (LASV) infection are estimated to occur in West Africa. Previous estimates of 100,000 to 300,000 annual infections are now considered likely to be an underestimate due to weak epidemiological surveillance systems, limited access to virological diagnostic tools, and the large number of asymptomatic or pauci‐symptomatic cases [3]. It is important to note that these estimates relate to LASV infections and not solely to clinical cases of Lassa fever. Approximately 80% of infections are thought to be asymptomatic, while around 20% progress to clinical forms that can sometimes be severe.

The overall case fatality rate is estimated at around 1%, but it can exceed 15% among hospitalized patients with severe forms of the disease, which constitutes a major public health problem in West Africa [3].

Furthermore, the virus’s high genetic diversity complicates the diagnosis in the absence of early specific symptoms and reduces the reliability of reverse transcriptase–polymerase chain 48 reaction (RT‐PCR) detection. Currently, there is no specific antiviral treatment for the LASV infection; however, ribavirin can inhibit viral replication during the early phase of illness, underscoring the critical need for timely diagnosis to save lives [6, 7].

Between 2011 and 2022, the Republic of Guinea reported 16 cases of Lassa fever, including 15 confirmed and one probable case [5], with a cross‐border case involving Liberia in 2018. An outbreak was also declared in 2019 in the forest region, with a confirmed LASV case by RT‐PCR [5].

On September 20, 2022, the Prefectural Health Directorate of N’Zérékoré was alerted by the Regional Directorate of the Hospital regarding the death of a confirmed Lassa fever case. A multidisciplinary investigation team conducted inquiries in healthcare facilities attended by the patients and within the family setting to identify the source of infection and list all potential contacts, thereby facilitating routine public health emergency management operations.

This case report describes the investigation of a confirmed Lassa fever case diagnosed late at the laboratory of the regional hospital in N’Zérékoré, following an interrupted and delayed clinical pathway, combined with inadequate clinical management. The findings provided valuable insights into the challenges of delayed case management and informed targeted public health interventions aimed at raising awareness and preventing outbreaks and resurgence.

## 2. Case Presentation

The patient was a 45‐year‐old male, employed as a logistician at the Catholic Organization for Human Promotion. He resided in the Gbangana neighborhood with his wife in a family compound and had traveled to the subprefecture of Bowé, in the southern part of Yomou Prefecture, prior to the onset of illness.

Between September 8 and 15, 2022, he developed intermittent fever accompanied by chest pain and oliguria. He initiated self‐medication at home using unidentified drugs, without any improvement in clinical symptoms. Subsequently, he sought care at Huguette Medical Clinic, where he received treatment consisting of Ringer’s lactate, paracetamol, and ceftriaxone. Referral to the regional hospital was recommended between September 15 and 16, 2022, due to treatment failure; however, the patient declined and instead consulted a traditional healer in Bowé South from September 16 to 18, receiving herbal decoctions.

On September 19, 2022, he returned to Huguette Medical Clinic with a clinical presentation of intermittent fever, chest pain, oliguria, severe physical asthenia, and hematemesis. He was immediately referred and transported in a private vehicle to the Regional Hospital, where he was admitted to the emergency department and underwent laboratory testing. Results were negative for Ebola and Marburg viruses. Consequently, other viral hemorrhagic fever was suspected, and the patient was transferred to the intensive care unit with decompensated anemia, evidenced by a hemoglobin level of 5.5 g/dL.

**FIGURE 1:**
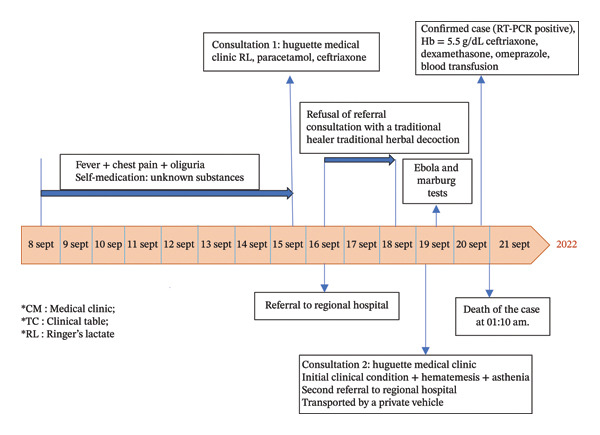
Therapeutic itinerary of the confirmed case in the urban commune of N’Zérékoré, Guinea, September 2022.

RT‐PCR testing confirmed Lassa fever infection on 19 September 2022. The patient was isolated at the Epidemic Treatment Center and received symptomatic treatment consisting of ceftriaxone, dexamethasone, omeprazole, and a blood transfusion, without ribavirin administration. The confirmed case died during the night of September 20‐21, 2022, at approximately 01:10 a.m (Figure 1).

The body was released to the family upon request, and burial was conducted without safety precautions, following usual funeral rites practiced in the urban commune of N’Zérékoré.

### 2.1. Conduct of the Investigation

On September 20, 2022, the Prefectural Health Directorate of N’Zérékoré was alerted by the Regional Hospital Directorate, as part of routine surveillance, of a confirmed case of Lassa fever originating from the Gbangana neighborhood within the Mohomou health area. The Prefectural Health Directorate convened an emergency meeting during which it was decided to establish a joint mission with technical and financial partners (TFPs) to support the N’Zérékoré health district in implementing response activities against Lassa fever.

**FIGURE 2:**
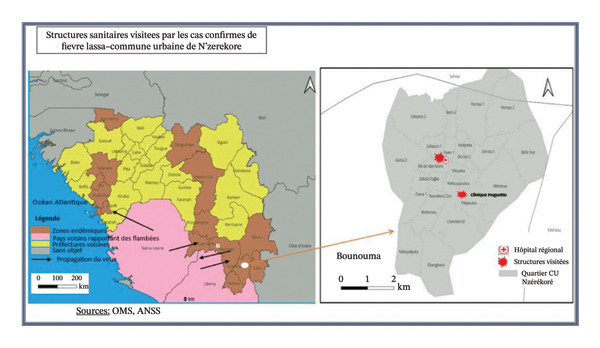
Map showing the urban commune of N’Zérékoré and the healthcare facilities visited by the confirmed Lassa fever case and investigation sites.

A comprehensive investigation was launched, leading to the identification of 36 contacts, including 19 healthcare workers from Huguette Medical Clinic and the Regional Hospital (Figure 2). The investigation team, made up of representatives from the National Agency for Health Security, the regional health authority, members of the district health management team of N’Zérékoré, and the World Health Organization (WHO), visited Huguette Medical Clinic, the Regional Hospital, the Epidemic Treatment Center, and the family of the confirmed case to collect information and compile a list of all potential contacts (Figure 2).

The investigation did not identify any case in which an epidemiological link could be established. However, confirmed cases of Lassa fever had previously been reported in the urban commune, specifically in the Mohomou neighborhood. Given the high population mobility, the proximity of neighborhoods, and the close living conditions, there is a significant likelihood of human‐to‐human transmission. Additionally, the presence of rodents within the household recognized vectors of Lassa fever transmission must be considered. These factors led to several hypotheses: The confirmed case may have been infected through (i) human‐to‐human transmission, (ii) direct contact with infected rodents, or (iii) indirect exposure via rodent excreta within the household environment.

In total, during the implementation of the investigation activities, 119 consultations were recorded in the visited health facilities, six alerts were reported concerning three living individuals and three deceased persons, 36 contacts were identified, listed, and monitored for 21 days, and six samples were collected from suspected cases. One of the six people tested returned a positive RT‐PCR result for Lassa fever despite showing no clinical symptoms. She was admitted to the Epidemiological Treatment Centre (CT‐Epi) in N’zerekoré, where she was placed in isolation and treated with ribavirin for 11 days. She was released after 21 days in isolation and following a negative RT‐PCR test, having shown no clinical symptoms. She was monitored postdischarge through home visits (weekly, then monthly) by health workers to check on her health. Subsequently, she was declared fully recovered, showing no clinical symptoms.

Finally, 24 samples were collected from rodents for environmental surveillance. The direct source of contamination for the confirmed case was not identified. However, all laboratory tests on the 24 samples came back negative.

## 3. Discussion

The investigation of the confirmed Lassa fever case in the urban commune of N’Zérékoré highlighted significant challenges in accessing appropriate healthcare services and systemic deficiencies that contribute to diagnostic delays in resource‐limited countries. In this particular case, the affected individual initially lost valuable time due to ineffective self‐medication before seeking formal healthcare services. Similar findings were reported in Benin by Anges Yadouleton and al. (2016), who described delays in the early detection and management of Lassa fever cases in health systems with characteristics comparable to those of Guinea [8]. Similar situations have also been observed in Nigeria, Sierra Leone (1970–1972), and Togo (2016) [9]. These delays may be explained by a lack of awareness of the initial clinical presentation of Lassa fever, which closely mimics common conditions such as malaria and influenza diseases that are easily treated and therefore, do not initially alarm patients [10–12]. Studies conducted in Nigeria (2020), Sierra Leone (2019), and Mali (2018) have also highlighted persistent challenges in the diagnosis and clinical management of viral hemorrhagic fevers [7, 13, 14]. Due to the nonspecific nature of the initial symptoms, diagnostic accuracy is often compromised, leading to delays in the implementation of life‐saving interventions. Financial constraints also exacerbate these delays, as many patients perceive the costs of biomedical care as unaffordable and turn first to alternative therapies. The confirmed case in our study experienced a further diagnostic delay after refusing transfer to a higher‐level healthcare facility despite worsening clinical symptoms. Instead, the patient sought treatment from a traditional healer before finally returning to the initial healthcare facility. Although this approach delayed access to appropriate biomedical care, traditional medicine remains a central aspect of healthcare‐seeking behavior in West Africa. Several studies conducted between 2020 and 2025 show that traditional healers are often the first point of contact for treatment due to their cultural acceptability, accessibility, and relatively low cost [3, 5, 15–17]. Rather than being viewed solely as an obstacle, traditional medicine could play a complementary role in disease surveillance and the early referral of patients if it were better integrated into national health systems. Strengthening collaboration between biomedical practitioners and traditional healers could thus help to reduce diagnostic delays and improve patient outcomes in endemic areas. Sidio et al. (2020) demonstrated that a significant proportion of the population in sub‐Saharan Africa, particularly in Côte d’Ivoire, uses traditional medicine as their primary source of care [18]. This preference, largely driven by cultural trust and accessibility, often delays recourse to formal medical facilities and thus compromises early diagnosis and treatment. The WHO estimates that around 80% of the population in sub‐Saharan Africa turns to traditional medicine as their first treatment option [19].

This situation reflects both a fear of the high costs of modern healthcare and a trust in traditional remedies, which are generally more accessible both financially and socially. The investigation in N’Zérékoré also revealed critical weaknesses in the healthcare system. For example, the Huguette Medical Clinic failed to arrange a safe medical transfer to the referral hospital, forcing the patient to make the journey in a private vehicle despite his critical condition, thereby increasing the potential risk of disease transmission. Furthermore, monotherapy with ceftriaxone was continued despite the patient’s clinical condition worsening. The lack of medical transport may partly explain the patient’s refusal to accept the initial referral and his subsequent report to traditional medicine. Furthermore, the preference for private facilities as the first choice of care is often driven by shorter waiting times and a better quality of care, although some of these facilities do not always have staff sufficiently trained to manage diseases with epidemic potential [20, 21]. Lassa fever was only suspected after negative test results for Ebola and Marburg, in the presence of hemorrhagic symptoms and severe decompensated anemia, 11 days after the onset of symptoms. Similar misleading clinical presentations, leading to diagnostic delays, have previously been reported in several investigations into Lassa fever [22, 23]. An in‐depth investigation conducted in the rural and urban communes of N’Zérékoré identified only three suspected cases meeting the operational definition of Lassa fever. Previous studies have reported comparable results, often attributed to the high mobility of exposed populations, their reluctance to participate in investigations, and the fragility of health systems [12, 24]. These factors complicate the issuance of alerts, the control of outbreaks, and the limitation of their geographical spread. The situation was also exacerbated by the absence of patients’ telephone contact details in some consultation records, as well as by the weakness of communication networks in certain localities. Our preliminary investigation identified 36 high‐risk contacts, including healthcare workers and family members who had been involved in the patient’s care. All contacts were monitored, and none showed any clinical abnormalities during the surveillance period. Although the number of contacts identified was lower than that recorded during the April 2022 outbreak in Koumassans (subprefecture of Tékolo) and Sidakoro (subprefecture of Kassadou), where 285 contacts had been identified, the follow‐up rate in our investigation reached 100%, compared with 92% during the previous outbreak. This improvement can be attributed to better adherence to standard operating procedures by health workers, as greater community adherence to epidemic response strategies. The three suspected cases isolated at the Epidemic Treatment Centre tested negative for viral hemorrhagic fevers at the N’Zérékoré Viral Hemorrhagic Fevers Laboratory and were subsequently referred to other departments for appropriate care. Environmental and epidemiological investigations led to the collection of 24 rodent samples, including organs, excreta, and saliva, all of which tested negative for viral hemorrhagic fevers. This outcome may reflect improved community sensitization and better adherence to preventive measures.

### 3.1. Strengths of the Investigation

The development of an integrated response plan and the regular coordination meetings facilitated the harmonization of stakeholders’ interventions and the establishment of a prefectural crisis committee in the affected health districts, aimed at mobilizing and engaging local authorities at the grassroots level.

The reporting of deaths and illnesses within families enables the control of certain disease risk factors. However, such control can only be effective with the involvement of all local stakeholders, including motivated and well‐informed community health workers, traditional healers who are often consulted by the population for health issues, local administrative authorities, elected officials, and community leaders. This inclusive approach fostered collaboration between response teams and communities, thereby achieving the expected outcomes.

The organization of community dialogs and educational sessions helped mobilize and engage all community actors in the response to Lassa fever and other epidemic‐prone diseases.

Overall, these combined interventions contributed significantly to breaking the chain of Lassa fever transmission in N’Zérékoré.

### 3.2. Limitations of the Investigation

The primary limitation was the inability to identify the source of contamination for the confirmed case. The reported cases did not allow for cross‐referencing epidemiological data to reconstruct the transmission chain. There were difficulties in tracing the index case and all contacts along its itinerary due to insufficient information. Furthermore, the environmental investigation could not access all rodent burrows and excreta. Public health limitations were also observed regarding the burial procedure of the confirmed case, as the hospital released the body to the family without any protective measures, despite confirmation of Lassa fever diagnosis by RT‐PCR. The body was transported across the city and buried according to usual funeral rites.

## 4. Conclusion

Health authorities should establish a systematic epidemiological investigation protocol for active case finding in both public and private health facilities and within communities, supported by an active and responsive surveillance system to detect cases and initiate investigations without delay. This protocol should incorporate environmental investigations through adequate collaboration with all stakeholders. Continuous training programs for healthcare personnel on epidemiological and environmental investigation methods and strengthening their capacity to apply the principles of antibiotic use in cases of persistent fever should also be implemented. Public and private health facilities should adopt infection prevention and control measures when managing febrile patients presenting with hemorrhagic signs. Moreover, it is imperative that political authorities and TFPs allocate the necessary resources to fund the development of Lassa fever response plans, conduct viral sequencing of the Lassa fever virus, and establish contingency plans for hemorrhagic fevers.

**Nomenclature**
CT‐Epi: Epidemic Treatment Center
LASV: Lassa virus
VHF: Viral hemorrhagic fevers
WHO: World Health Organization
TFP: Technical and financial partner
RT‐PCR: Reverse transcription polymerase chain reaction
SOP: Standard operating procedure

## Author Contributions

The protocol was drafted by I.S.C. with the contribution of M.A.D. and validated by A.A.K., S.D., M.O.B., S.C., F.C., M.M., F.M.K., A.L., and L.C. I.S.C., M.A.D., A.A.K., A.L., and L.C. participated in the investigation. I.S.C. prepared the investigation report and conducted the literature reviews. I.S.C. and M.M. analyzed the data. E.M.L.E. conducted the literature review, drafted the initial version, and made major corrections to the manuscript. I.S.C., L.L.K., and D.O.D.K. contributed to manuscript revision.

## Funding

No funding was received.

## Disclosure

I.S.C., E.M.L.E., L.L.K., D.O.D.K., M.A.D., S.D., M.O.B., S.C., and F.M.K. read and approved the final manuscript.

## Ethics Statement

This study was approved by the local ethics committee of the Regional Hospital of the urban commune of N’Zérékoré (No. 73). The investigation was conducted anonymously and confidentially. Contact tracing was carried out by the Epidemiological Surveillance Unit of the N’Zérékoré health district. All contacts provided oral consent to participate in the follow‐up. Data collected during contact monitoring were treated confidentially and anonymously. Furthermore, the investigation team obtained authorization from the health authorities (Prefectural Director and Regional Health Inspector) of the N’Zérékoré region, prior to data collection.

## Consent

Written informed consent for publication of the case was obtained from the patient’s elder brother (relative).

## Conflicts of Interest

The authors declare no conflicts of interest.

## Data Availability

The patient’s medical record is available from the corresponding author upon reasonable request.
